# Duration of Protection From Pneumonia After Pneumococcal Vaccination in Hemodialysis Patients (DOPPIO): Protocol for a Prospective Multicenter Study

**DOI:** 10.2196/45712

**Published:** 2023-07-12

**Authors:** Sibylle Mellinghoff, Gero von Gersdorff, Caroline Bruns, Kerstin Albus, Vassiliki Dimitriou, Angela Steinbach, Mathias Schaller, Jörg Janne Vehreschild, Oliver A Cornely, Blasius Janusch Liss

**Affiliations:** 1 Centre for Integrated Oncology Aachen Bonn Cologne Duesseldorf and Excellence Centre for Medical Mycology Department I of Internal Medicine Faculty of Medicine and University Hospital Cologne, University of Cologne Cologne Germany; 2 German Centre for Infection Research, Partner Site Bonn-Cologne Cologne Germany; 3 Faculty of Medicine and University Hospital Cologne Translational Research, Cologne Excellence Cluster on Cellular Stress Responses in Aging-Associated Diseases University of Cologne Cologne Germany; 4 Faculty of Medicine and University Hospital Cologne Department II of Internal Medicine – QiN-group University of Cologne Cologne Germany; 5 Kuratorium fuer Haemaodialyse (KfH)-Board of Trustees for Dialysis and Kidney Transplantation Neu-Isenburg Germany; 6 Translational Research, Cologne Excellence Cluster on Cellular Stress Responses in Aging-Associated Diseases Faculty of Medicine and University Hospital Cologne University of Cologne Cologne Germany; 7 Clinical Trials Centre Cologne (ZKS Köln) Faculty of Medicine and University Hospital Cologne University of Cologne Cologne Germany; 8 Department I of Internal Medicine Helios University Hospital Wuppertal Wuppertal Germany; 9 School of Medicine Faculty of Health Witten/Herdecke University Witten Germany

**Keywords:** Streptococcus pneumoniae, pneumococcal, vaccination, hemodialysis, clinical trial, vaccine, pulmonary, pneumonia, infectious disease, communicable disease, dialysis, kidney

## Abstract

**Background:**

Pneumonia is a leading cause of death in patients with end-stage chronic kidney disease treated with dialysis. Current vaccination schedules recommend pneumococcal vaccination. However, this schedule disregards findings of rapid titer decline in adult hemodialysis patients after 12 months.

**Objective:**

The primary objective is to compare pneumonia rates between recently vaccinated patients and patients vaccinated more than 2 years ago. As an exploratory objective, antipneumococcal antibody titers in hemodialysis patients will be determined as a function. Factors influencing antibody kinetics will be identified.

**Methods:**

Within this prospective multicenter study, we aim to compare 2 strata of vaccinated patients: those recently vaccinated and those vaccinated more than 2 years ago. A total of 792 patients will be enrolled. Twelve partner sites (within the German Centre for Infection Research [DZIF]) with allocated dialysis practices participate in this study. All dialysis patients who are vaccinated against pneumococcal infection in accordance with Robert Koch Institute guidelines prior to enrollment will be eligible. Data on baseline demographics, vaccination history, and underlying disease will be assessed. Pneumococcal antibody titers will be determined at baseline and every 3 months for 2 years. DZIF clinical trial units coordinate titer assessment schedules and actively follow-up on study patients for 2-5 years after enrollment, including validation of end points of hospitalization, pneumonia, and death.

**Results:**

The study has enrolled 792 patients and the last follow-up has been completed. Currently, the statistical and laboratory analyses are ongoing.

**Conclusions:**

Results will increase physician adherence to current recommendations. Establishing a framework for the efficient evaluation of guideline recommendations through a combination of routine and study data will inform the evidence base for future guidelines.

**Trial Registration:**

ClinicalTrials.gov NCT03350425; https://clinicaltrials.gov/ct2/show/NCT03350425

**International Registered Report Identifier (IRRID):**

DERR1-10.2196/45712

## Introduction

### Background

The global burden of disease continues to shift from acute communicable to chronic noncommunicable diseases and from years of life lost to years lived with disability [1]. Advanced chronic kidney disease (CKD; stages 3, 4, and 5 out of 5) is among the most common chronic health conditions in Germany [2]. Among CKD 5D (chronic kidney disease stage 5 with an indication for dialysis) patients, acute infections remain a leading cause of morbidity, mortality, and costs [3]. The most common infectious disease in CKD 5D patients is vaccine-preventable pneumonia. Recommendations in current vaccination guidelines acknowledge this substantial risk [4,5].

Observational studies suggest that pneumococcal vaccines lower mortality and morbidity in CKD 5D patients [6,7]. While a recent meta-analysis demonstrated the efficacy of pneumococcal vaccination in reducing pneumonia in the overall adult target population [8], randomized or well-designed prospective studies in a hemodialysis population are still lacking. Prior immunological studies in hemodialysis patients have been limited by low patient numbers and mixed populations (CKD 1-4, hemodialysis, and renal transplant recipients) [9]. While these studies suggest good immunogenicity of the vaccines, no attempts were made to verify the relevance of titers toward clinical end points in this population.

Current vaccination schedules recommend pneumococcal vaccination for patients older than 60 years or at increased risk for invasive pneumococcal disease every 6 years [4,5]. However, this schedule ignores preliminary findings of rapid titer decline in hemodialysis patients. Specifically, titers seem to decline substantially after 6 to 12 months in adult dialysis patients [10]. A limited number of studies with a longer follow-up period suggest a possible benefit of revaccination after 2 years [11,12].

### Scientific Rationale

Pneumonia is a leading cause of death in patients with end-stage chronic kidney disease (stage 5) treated with dialysis (CKD 5D). Acknowledging that many infectious complications are vaccine-preventable, specific recommendations have been issued for patients at risk.

In CKD 5D patients, these recommendations are based on weak evidence, namely immunologic studies [9] demonstrating the induction of antibody production and retrospective cohorts compare vaccinated to unvaccinated patients. Until today, not a single prospective study demonstrating a clinical benefit of pneumococcal vaccination in hemodialysis patients has been published. Furthermore, the aforementioned immunological studies suggest a rapid decline of protective antibodies in such patients, calling the current Robert Koch Institute (RKI) recommendation of vaccinating every 6 years into question.

While further research into the efficacy of pneumococcal vaccinations in hemodialysis patients is required, withholding vaccines would be unethical. However, comparing vaccinated patients to patients unwilling or unable to be vaccinated is likely to be a biased approach. We will thus compare 2 strata of vaccinated patients—those recently vaccinated and those vaccinated more than 2 years ago—concerning their protective antibody titers and their rates of pneumonia. We hypothesize that patients vaccinated more than 2 years ago, while in line with the current recommendation, no longer possess sufficient antibody response (<0.35 µg/mL serotype-specific IgG as defined in the World Health Organization [WHO] recommendation [13]) and thus have a risk of pneumonia comparable to an unvaccinated patient. Simultaneously, we will analyze titers of protective antibodies over time and identify factors predicting a more rapid decline.

### Objectives

#### Primary Objective

The primary objective is to compare pneumonia rates between newly vaccinated hemodialysis patients and those vaccinated against pneumococcal infection more than 2 years ago.

#### Explorative Objectives

The explorative objectives are as follows:

- To describe the pneumococcal antibody titers in hemodialysis patients as a function of time since vaccination
- To determine factors influencing antibody kinetics (eg, age, prior or ongoing immunosuppression, medication, and prior vaccination history)
- To explore the relationship between antipneumococcal antibody titers and the incidence of pneumonia in hemodialysis patients and to extrapolate a possible cutoff for protection from pneumonia
- To evaluate the role of patients’ history (eg, underlying kidney disease) and clinical course (eg, prior hospitalizations) on pneumonia and vaccine response

## Methods

### Study Design

This is a prospective, multicenter, noninterventional study of vaccine efficacy in hemodialysis patients. All dialysis patients who are vaccinated against pneumococcal infection in accordance with RKI guidelines before enrollment will be eligible. Eligibility depends on the agreement of signing an informed consent allowing for data entry into the study electronic case report form (eCRF) and participation in the electronic quality registry, thus enabling data transfer from the patient’s Kuratorium für Dialyse und Nierentransplantation e.V. (KfH) electronic health record into the eCRF, titer assessments, and active follow-up observation. A minimum of 792 patients will be enrolled.

Participating sites will screen all hemodialysis patients at their outpatient dialysis clinic for eligibility. Eligible patients will be approached and informed by their physicians. If a patient agrees to participate in the study and signs an informed consent form (ICF), basic data regarding vaccination history, underlying disease, and so on will be entered or imported electronically into the study eCRF. Pneumococcal antibody titers will be drawn at baseline and every 3 months. For newly vaccinated patients, baseline titers will be drawn 4 weeks after vaccination. Deutsches Zentrum für Infektionsforschung (DZIF; English: German Center for Infection Research) clinical trial units (CTUs) will coordinate titer assessment schedules and inform the participating physicians of upcoming blood samplings. The samples will be taken on dialysis days from an already inserted-intravascular catheter. DZIF CTUs will actively follow up on study patients for 2-5 years after enrollment. This follow-up will include validation of all hospitalizations and deaths documented in the electronic registry to assess whether the primary end point (all-cause pneumonia) occurred. During months 25-30, only the primary end point will be recorded (ie, no blood samples will be taken). Additionally, dialysis units will be contacted every 6 months to assess if pneumonia occurred that did not require hospitalization. Blood samples will be stored frozen at DZIF CTUs, and titers will be analyzed at the end of this study. All therapies and diagnostics, including vaccinations, will be administered solely as part of the clinical routine and as recommended by appropriate guidelines (eg, RKI Ständige Impfkommission [STIKO] recommendations). Thus, Arzneimittelgesetz (AMG) does not apply to this study, and participating physicians do not require Good Clinical Practice (GCP) certification.

### Data Collection

Study nurses will enter data into the eCRF either directly or by transfer of an extract from the patient’s electronic registry file (Qualität in der Nephrologie [QiN]; English: Quality in Nephrology database). The registry will also alert the study nurse to events during follow-up such as hospitalization, death, and transfer out of the dialysis unit, which are reliably captured because they are needed for billing purposes.

The central data repository will be the eCRF. Source documents will be stored in each patient’s study file.

### Study Procedure

In principle, all KfH dialysis practices will be eligible to participate in this study. KfH practices can be considered representative of the German dialysis population since they are widely distributed throughout Germany and about 20%-25% of all hemodialysis patients are treated there. Site selection will depend on the willingness of the attending physicians to participate and key characteristics of the practice (eg, site location and number of patients). Criteria for site selection will be developed by QiN-group and DZIF-Köln. A number of suitable KfH dialysis practices will be allocated to each of the 12 DZIF CTU sites: Universitätsklinikum Bonn, Medizinische Klinik Borstel, Universitätsklinikum Eppendorf, Universitätsklinikum Gießen und Marburg, Standort Gießen, Medizinische Hochschule Hannover, Universitätsklinikum Heidelberg, Universitätsklinikum Köln (AöR), Universitätsklinikum Schleswig-Holstein/Campus Lübeck, Universitätsklinikum Gießen und Marburg, Standort Marburg, Klinikum der Universität München, Technische Universität München, and Universitätsklinikum Tübingen.

QiN-group will assist DZIF CTU personnel with site selection and internal KfH study participation formalities. DZIF CTU personnel will cooperate with the KfH physicians to assist in setting up subject prescreening and informed consent procedures. Informed consent will be obtained by treating physicians.

The sample size has been calculated to demonstrate a significantly lower all-cause community-acquired pneumonia (CAP) rate in newly vaccinated patients compared to patients vaccinated more than 2 years before enrollment.

The incidence of pneumonia in hemodialysis patients was reported as 29.0/100 patient-years (py) [14]. Data on the efficacy of pneumococcal vaccines for preventing pneumonia in hemodialysis patients is lacking. However, a recent meta-analysis demonstrated pneumococcal vaccination to result in a relative risk for pneumonia of 0.72 (95% CI 0.69-0.94) in an immunocompetent target population (ie, adults at risk or >65 years) [8].

In general, immunological studies in hemodialysis patients have been carried out with limited patient numbers and in mixed populations (CKD, hemodialysis, and renal transplant recipients) [9]. Most studies suggest good initial immunogenicity, but titers seem to significantly decline after 6 to 12 months in adult dialysis patients [10]. The limited number of studies with longer follow-up periods suggests a possible benefit of revaccination after 2 years [11,12].

For the purpose of this calculation, we assume protection from pneumonia in the first 2 years to be equal to the protection recently demonstrated in an adult immunocompetent target population (relative risk 0.72) and afterward return to baseline. Thus, expected rates of pneumonia are 29.0/100 py in patients vaccinated >2 years ago and 20.88/100 py in patients vaccinated <2 years ago, respectively. To demonstrate the superiority of a more recent vaccination in preventing pneumonia with α=.05 and (1–β)=0.80, we would thus require 2 groups of 442 patients each (884 patients overall). Furthermore, the rate of pneumonia is also likely to be greater in an underprotected population than seen in the overall hemodialysis population, which includes a relevant number of freshly vaccinated patients. Additionally, the possibility of an unbalanced distribution of recently vaccinated patients (in a ratio of up to 1:7) was considered. A first statistical analysis will be discussed in an interim study report after 18 months.

### Amendment After Interim Study Report

After an enrollment period of 18 months, which had been extended twice, 710 patients had been included in the study and had a fully documented vaccination status, 174 less than planned. 596 had been vaccinated less than 2 years ago, and 114 had been vaccinated 2 or more years before enrollment, respectively. This represents a ratio of 1:5, which was well within the range of an unbalanced patient distribution, which had been explored in the sample size calculation. Assuming that further recruitment would continue at this ratio and that risk reduction remained at 0.72, 792 patients were then considered sufficient if the observation period was extended to 2.5 years. These assumptions yielded a power (1–β) of 0.81 while keeping the type-I error at α=.05.

### Eligibility Criteria

#### Inclusion Criteria

The inclusion criteria included:

1. Patients who signed ICF, including enrollment in the registry database (QiN-registry)
2. Patients with stage 5 CKD treated with chronic hemodialysis
3. Patients who are vaccinated against pneumococcal infection in accordance with current STIKO recommendations
4. Patients who were 18 years or older

#### Exclusion Criteria

The exclusion criteria included patients unwilling or ineligible for vaccination against pneumococcal infection under current STIKO recommendations.

A subject may withdraw from the clinical trial at any time for any reason without consequences. Discontinuation from the study will have no influence on the availability of vaccination for that patient. In case of premature discontinuation, the reason for discontinuation should be collected, but it is not obligatory. In the event of withdrawal, all blood samples will be destroyed according to data protection requirements. Data cannot be removed from already-completed studies or scientific analyses. A subject who discontinues the trial will not be replaced.

### Enrollment of Study Subjects

**Figure 1:**
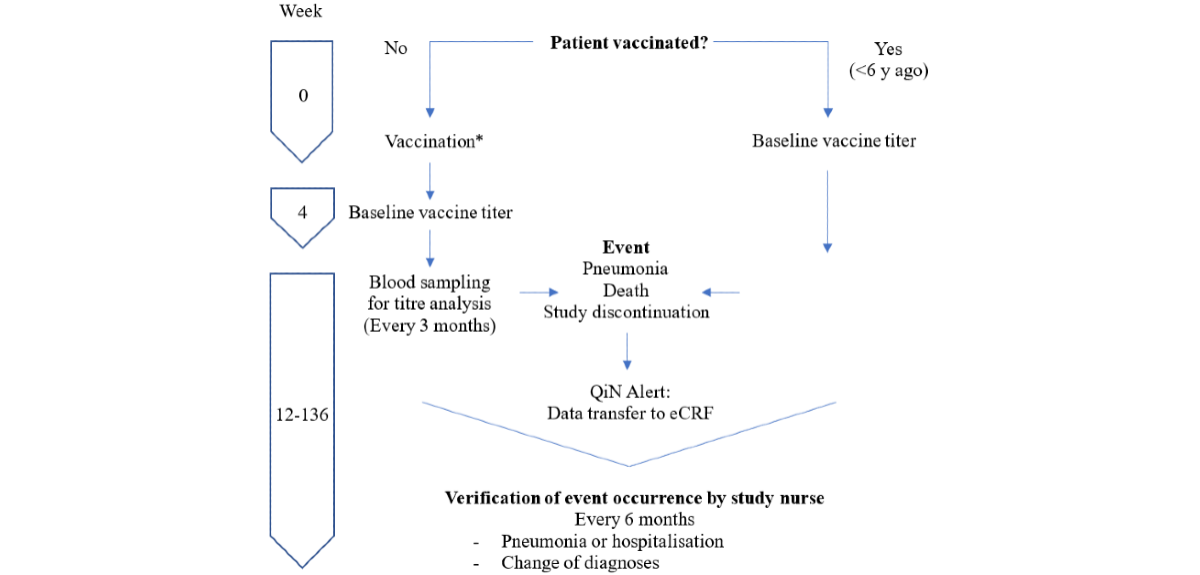
Schedule of vaccine titer assessments in the course of the Duration of Protection From Pneumonia After Pneumococcal Vaccination in Hemodialysis Patients (DOPPIO) study. *Decision for or against vaccination will be based upon the advice of the treating physician previously (along STIKO [Ständige Impfkommission]–criteria) and is not related to DOPPIO. eCRF: electronic case report form; QiN: quality in nephrology.

Eligible study subjects will be identified by their dialysis physician. If the subject does satisfy eligibility criteria and signs the ICF, he or she will be enrolled into the Duration of Protection From Pneumonia After Pneumococcal Vaccination in Hemodialysis Patients (DOPPIO) study by entering their data into the enrollment log and registering the subject in the eCRF. Basic data regarding vaccination history, underlying disorders, and so on will be captured directly in the study eCRF or transferred into the eCRF from the QiN database. The study’s conduct is illustrated in Figure 1.

Blood samples for pneumococcal antibody titer analysis will be drawn at baseline and every 3 months (with a range of 6 days). For newly vaccinated patients, baseline titers will be drawn 4 weeks after vaccination (Figure 1). A total of 6750 blood samples from 789 study subjects will be collected and analyzed.

DZIF CTU study nurses will coordinate titer assessment schedules and inform participating physicians of upcoming blood samplings. Samples will be drawn over an existing venous or arteriovenous access before dialysis and thus constitute only a minimal additional burden for both patients and physicians. All titers will be analyzed at the end of the study. An enzyme immunoassay will be performed to detect the presence of pneumococcal antibodies.

### Management of Samples

For all subjects, 9 mL of blood will be collected in a 9-mL tube provided by the sponsor. Samples will be taken every 3 months after the baseline sample. For newly vaccinated patients, baseline samples will be taken 4 weeks after vaccination.

Immediately prior to the blood draw, the staff member performing the procedure will verify the subject’s identity. A label will be attached to the tube. The tube will be centrifuged within 3 hours after sampling. The tube will be shipped to the allocated DZIF site and frozen within 24 hours. Subject number, date of sampling, number of aliquots, date and time of preparation, and the subject’s consent are documented in a sample identification list at the KfH dialysis practices and recorded as source data. Samples will be stored at DZIF CTUs and sent frozen in 1 batch to the laboratory at the end of the study. Results will be transferred electronically to the study nurses at the end of the study for recording into the eCRF.

### Data Management Plan

The data will be recorded securely and electronically. The data are the sole property of the sponsor and must not be made available in any form to third parties, except for authorized sponsor’s representatives or appropriate regulatory authorities, without written permission from the sponsor. Data obtained from KfH via QiN becomes the property of the sponsor, as specified in the agreement between KfH and DZIF. Only data from enrolled patients will be transferred. The investigator will ensure that all data are entered legibly, completely, accurately, and conform to source documents.

The investigator will review and approve data, with the investigator’s validation serving as attestation of the investigator’s responsibility for ensuring that all data are complete, accurate, and authentic.

All information obtained during the study will be recorded digitally in conformity with the applicable laws and regulations.

### Ethics Approval

#### Overview

The study was approved by the lead Ethics Committee of the Medical Faculty of the University of Cologne (Cologne, Germany; No. 17-274) and subsequently received secondary approval by the 11 Ethics Committees of the respective DZIF CTU sites (ref. DZIF CTU site list, section study procedure).

The study was further approved by the Paul-Ehrlich-Institut (PEI), Federal Institute for Vaccines and Biomedicines, as a noninterventional study (NIS401) and registered at ClinicalTrials.gov with the identifier NCT03350425.

The study will be performed in accordance with all applicable laws and regulations, including the International Conference on Harmonization Guideline for Good Clinical Practice (GCP), the ethical principles that have their origins in the Declaration of Helsinki (current official version: World Medical Association [15]), and applicable privacy laws such as national laws and the General Data Protection Regulation, which has been in effect since May 25, 2018. The minimum required deidentified personal data will be captured in an electronic database, and any publication of study results will be anonymized, only containing summarized statistical analyses.

#### Subject-Informed Consent

KfH physicians will assist in setting up subject prescreening and informed consent procedures. Informed consent will be obtained by the treating physicians. The ICF will be signed prior to any study activities by the subject and treating physician conducting the informed consent process. No compensation is foreseen for study participants.

### Study Conduct and Statistical Analyses

The primary objective of this study is to compare pneumonia rates and outcomes between both groups. To achieve this, the raw number of events and raw rates of events per 100 patient years will be computed. For each group, the mean, median, SD, IQR, and minimum and maximum of demographical variables like age and sex will be computed. These observed variables will be used to build a Cox proportional hazard model. If applicable, this model will incorporate time-varying covariates and consider recurring events. To quantify the expected protective effect of a high titer level, multiple functional relationships will be explored, including but not limited to linear, quadratic, and step functions. Step functions will be created by dividing the titer levels into 10 mutually exclusive strata and estimating each risk individually. If possible, the largest difference between 2 neighboring strata will be identified as a threshold for an adequate titer level.

The secondary objectives of this study are to describe antibody titers as a function of time and factors influencing antibody kinetics. Descriptive statistics for titer levels at all intervals will be computed for absolute values as well as relative values (relative to the first observed or personal maximum). Levels for patients grouped by age, sex, and other variables of interest will be calculated. To allow a comparison, the mean, median, SD, IQR, and minimum and maximum titer levels will be calculated.

To identify which factors influence the absolute antibody titer level, multiple multivariate models will be built. In patients with available titers, risk factors for insufficient protection will be determined by multiple regression analyses of data including vaccination history and timing, medication exposure since last vaccination, history of underlying kidney disorder, dialysis, and demographic factors.

The titer levels will be assessed graphically by comparing boxplots of the titer levels at different time intervals. Concurrent titer levels will be tested for statistically significant differences by using a *t* test or a Mann-Whitney U test, whichever is applicable.

Differences in hospitalization as well as vaccination rates between KfH centers will be studied. QQ plots and tests for normal distribution will be performed on the observed hospitalization rates. Vaccination rates will be studied for outliers. For all factors used in model building, a region of common support will be established.

## Results

The study has enrolled 792 patients and the last follow-up has been completed. Currently, the statistical and laboratory analyses are ongoing.

## Discussion

### Overview

Patients on dialysis are among the best documented for any chronic condition in outpatient medicine. This prospective study is among the first to capitalize on that and to demonstrate the integration of routine data and study data into a single data set. This framework for the efficient evaluation of guideline recommendations will form the evidence base for future guidelines and will increase physician adherence to current recommendations.

### Principal Results

Data on baseline demographics, vaccination history, and underlying disease will be assessed. Pneumococcal antibody titers will be determined at baseline and every 3 months for 2 years. DZIF CTUs coordinate titer assessment schedules and actively follow up on study patients for 2 years after enrollment, including validation of hospitalization, pneumonia, and death. We will analyze antibody titers over time and identify factors influencing antibody kinetics. In addition, the relationship between antibody titers and the incidence of pneumonia in hemodialysis patients will be investigated, and a possible cutoff for protection from pneumonia will be extrapolated.

### Limitations

Our study has several limitations. The study is conducted during the SARS-CoV-2 pandemic, which leads to interruptions in observation (especially serologically) and a novel cause of pneumonia. In addition, the study does not investigate the causative pathogen of pneumonia. However, *Streptococcus pneumoniae* has historically been the most common cause of pneumonia, by far [16], and remains the most common bacterial cause of CAP, leading to the hospitalization of adults today. In the United States, 5 to 15 percent of CAP cases have been attributed to *S pneumoniae* [17-19]. More recent comprehensive molecular testing of lower respiratory tract specimens indicates that this is an underestimate and suggests more than 35% of cases may be due to *S pneumoniae* [20]. This percentage has been proven to be even higher in Europe [21,22]. Finally, the study was performed in a single country with a homogeneous population of participants, which limits generalizability to other populations.

Nonetheless, we believe that the study is suited to answer the clinically important question of the duration of antipneumococcal vaccination.

### Conclusions

Current vaccination schedules recommend pneumococcal vaccination for CKD-5D patients every 6 years. However, this timing disregards findings of rapid titer decline in adult hemodialysis patients after 6 to 12 months. By comparing pneumonia rates between recently vaccinated patients and those who were vaccinated more than 2 years ago, we may estimate the duration of protection by antipneumococcal vaccination. In addition, antipneumococcal antibody titers in hemodialysis patients will be determined as a function of time since vaccination and provide further conclusions.

Through its conduct, this prospective study will increase physician adherence to current recommendations. Establishing a framework for the efficient evaluation of guideline recommendations through a combination of routine and study data will inform the evidence base for future guidelines.

## Abbreviations

CAP: community-acquired pneumonia
CKD: chronic kidney disease
CTU: clinical trial unit
DOPPIO: Duration of Protection From Pneumonia After Pneumococcal Vaccination in Hemodialysis Patients
DZIF: Deutsches Zentrum für Infektionsforschung (German Center for Infection Research)
eCRF: electronic case report form
ICF: informed consent form
KfH: Kuratorium für Dialyse und Nierentransplantation e.V.
QiN: Qualität in der Nephrologie (Quality in Nephrology)
RKI: Robert Koch Institute
STIKO: Ständige Impfkommission

## Data Availability

Data will be available as publication after finalization of the study.
